# Scientific Mobility, Training and Entrepreneurial Skills in Health Sciences: The Spanish Case

**DOI:** 10.3390/ijerph18042195

**Published:** 2021-02-23

**Authors:** Pedro Aceituno-Aceituno, Joaquín Danvila-del-Valle, Abel González García, Carlos Bousoño-Calzón

**Affiliations:** 1 Department of Business Administration and Management and Economics, Madrid Open University (MOU), Collado Villalba, 28400 Madrid, Spain; joaquin.danvila@udima.es; 2 Department of Criminology, Madrid Open University (MOU), Collado Villalba, 28400 Madrid, Spain; abel.gonzalez@udima.es; 3 Department of Signal Theory and Communications, Carlos III University of Madrid (UC3M), Leganés, 28911 Madrid, Spain; cbousono@tsc.uc3m.es

**Keywords:** Health Sciences, researchers, entrepreneurship, intrapreneurship, training, skills, transfer, mobility, entrepreneurial intentions

## Abstract

The activity of scientists promotes medical research in health services. However, on many occasions, these professionals do not know how to transfer their research results to the market. Therefore, it is worth providing data on aspects such as training in entrepreneurship and scientific mobility to foster knowledge transfer. This paper discusses data on the Spanish case in Health Sciences to devise effective policies in these areas. To this end, following the methodology of the Global Entrepreneurship Monitor report and the existing scientific literature, 291 researchers involved in scientific mobility in Spain have been interviewed. Of these, 90 belonged to health areas: Spanish scientists abroad (37), Scientists returned to Spain (16), and Young researchers in Spain (37). The results show that the mobile scientists in this area have more entrepreneurial and intrapreneurial intentions, have acquired more entrepreneurial skills, and have received more training in these subjects. Furthermore, there are few permanent positions for all these groups whose mobility decisions fundamentally depend on job opportunities, so the health authorities can intensify these measures to promote knowledge transfer.

## 1. Introduction

Research and innovation are crucial to improving the provision of health care in national health systems [1,2,3,4], and their results can turn into products or processes that create economic value [5] and employment [6]. In this regard, health institutions such as hospitals fulfill these functions when, in addition to assuming the functions of care, teaching, and research [7,8], they undertake a fourth mission associated with innovation and transfer, in which the results can be transferred and contribute to economic and social development, making them entrepreneurial hospitals [5,9,10,11].

In view of these important functions, the work of scientists is a strong stimulus for medical research in the health services [12,13], and, in addition, their performance is improved by their involvement in translational research [14]. However, health professionals are often unaware of how the product will create economic value [15], and therefore it is important for these professionals to acquire entrepreneurial skills that will enable them to transfer their results to the market [16].

The government and universities have attached increasing importance to entrepreneurship, in parallel with the economic transformations which have taken place in the last third of the 20th century [17], and it is now a priority on political agendas throughout the world as a tool to promote economic growth, generate employment and create social capital [18]. This trend promoted policies based on training in entrepreneurial subjects [19] and has made this type of education a fundamental function of universities [20,21], especially at times such as the current Covid-19 pandemic, when the entrepreneurial intentions of university students are diminishing [22]. This positive view of entrepreneurship training is endorsed [23,24,25,26], as it creates positive stimuli and increases the entrepreneurial intention of students [27,28,29]. Nevertheless, there are also authors for whom the effects of entrepreneurship training are not well known or consistent [30] because it is difficult to assess the effect of this type of entrepreneurship education [31]. Other authors go even further and consider this type of training to be ineffective in increasing entrepreneurial intentions [32,33].

In the case of researchers, international mobility promotes entrepreneurship [34,35], because carrying out their activity in new, highly technological environments increases their possibilities of finding new business opportunities [36]. In addition to promoting entrepreneurship, the knowledge acquired from international experience helps access economic resources, networks with a high entrepreneurial culture, the influence of academic entrepreneurs, and above all, the development of entrepreneurial skills [37], which is vital for the transfer of knowledge in Health Science research.

Therefore, it is important for researchers to return from their mobility abroad or, at least, to collaborate with their home country [38,39,40], since individuals who have worked in many different environments are more likely to be aware of opportunities to compete [41], carry out knowledge transfer activities [42,43] and obtain support for the commercialization of their findings [44,45]. This background makes it easier for researchers who have moved abroad to help their organizations increase their patent applications [46,47,48,49,50], publications [50,51,52,53] and impact [54], by overcoming the difficulties of transfer and the mismatch between scientific knowledge and its commercial application [55], which prevents this knowledge from becoming economically useful [56].

However, the number of companies created by researchers is relatively low [37,57,58], but their role in other entrepreneurial activity such as intrapreneurship is very important. This last activity can be defined as a process undertaken by a person involved in the leadership and development of an entrepreneurial initiative for his organization; examples are the creation of a new product or service, a new company, or a new business unit [59]. The World Economic Forum (WEF) in collaboration with the Global Entrepreneurship Monitor (GEM) [60] has carried out a survey of the adult population which shows that countries with more intrapreneurs create more jobs and are more competitive than those with more entrepreneurs. In the latter case, each percentage point increase in competitiveness is associated with a 2.5% increase in the rate of intrapreneurs. Therefore, it is important to attract researchers who have moved abroad with an entrepreneurial profile.

When it comes to attracting these scientists, the primary reasons for returning to their country of origin are personal, but despite this, these countries do have certain possibilities of establishing measures to encourage their return, since the decision of their researchers also depends in part on the job opportunities created in their own countries, although it is difficult to keep track of them abroad once they have left the country [61].

In these aspects of scientific mobility, any country needs to know the number of its researchers working abroad. In Spain, this data is not known, and attempts to establish this type of census have achieved only partial results [62,63,64,65]. In addition, in its National Health System (NHS) the transfer of knowledge generated by research has been limited [66], with hospitals not playing a major role in innovation [9,10,67], so increasing these aspects continues to be an important goal, as shown by the most recent research, development and innovation (R&D&I) plans and strategies [68,69,70,71,72,73].

Likewise, various surveys conducted for the health area [74,75] show that the decision of Spanish researchers to return depends to a large extent on whether they are offered job opportunities, so the health authorities and administrators have the possibility of establishing measures to encourage this return. These measures could also be accompanied by others to ensure that these researchers at least collaborate with Spanish science, as the degree of an international collaboration of these researchers is quite high compared to the lower level of collaboration with national scientific institutions [76].

Finally, in relation to the entrepreneurship of Spanish scientific mobility, the aforementioned study [58] shows that scientific mobility does indeed encourage entrepreneurship, and especially intrapreneurship and the development of entrepreneurial skills, so, in view of the aforementioned importance of these skills, it may be worthwhile to gain a more in-depth understanding of this development in the health sector in the Spanish case.

For all the above reasons, this paper aims to provide and discuss data on the training received in entrepreneurship by Spanish scientific mobility workers in Health Science, their acquisition of entrepreneurial skills, and the encouragement of their entrepreneurial and intrapreneurial intentions, so that effective training and scientific mobility policies can be developed to promote entrepreneurship in these areas in the interests of greater economic and social development.

## 2. Materials and Methods

To fulfill the objective of this paper, a quantitative methodology has been selected based on a descriptive study [77], supported by data on the acquisition and development of entrepreneurial skills for Spanish scientific mobility. Within this group, the following categories have been taken into account:

(1) Spanish scientists abroad (SSA).
(2) Scientists who have returned to Spain after carrying out their scientific activity abroad for at least one year (Spanish Returned Scientists) (SRS).
(3) Young researchers working in Spain (YRS), are highly likely to go abroad in search of the differential advantages, which are those that will enable them in the future to increase their differences with other scientists who do not have such advantages. This concept, discovered by Merton [78], is based on the following biblical passage from the Gospel according to St Matthew, and also from St Mark: “For unto every one that hath shall be given, and he shall have abundance: but from him, that hath not shall be taken away even that which he hath”. According to Merton [79], these advantages are the following: training, funding, the prestige of the host institution, excellent teamwork, and research career. The most decisive variables for the progress of this group in Health Sciences in Spain are focused on these differential advantages [75]. The definition of this group is that of researchers who have started doctoral courses in Spain and have continued to carry out scientific work until the age of 41.

### 2.1. Ethics Statement

This survey has been approved by the Ethics Committee of Madrid Open University (MOU; Project identification code: INNOVACEF 2019/2021; dated 21 November 2018). In the questionnaire itself, all participants gave their informed consent to participate in the survey. The completion of the questionnaire was voluntary and anonymous. The authors of this paper did not interact with the respondents. The data can be found in the tables. Researchers who wish to access any type of information from the survey can contact the authors of the paper, provided they respect the anonymity of the participants.

### 2.2. Population and Sample

In the latest R&D&I plans and strategies [68,69,70,71,72,73], as described above, the funding of the international mobility of researchers from all areas of knowledge in the Spanish System of Science, Technology, and Innovation (SECTI) has been proposed, for them to acquire training in new knowledge and skills. Therefore, in addition to analyzing the figures regarding scientists in health areas, it is also important to make a comparison between researchers from different areas of knowledge to determine possible differences in training in entrepreneurship, the acquisition of entrepreneurial skills, and entrepreneurial and intrapreneurial intentions associated with good practices in certain areas, which can be applied to improve training and scientific mobility in health areas.

It is difficult to keep track of researchers abroad, as stated above [61]. Likewise, it was also previously mentioned that in Spain various attempts have been made to take a census of these researchers which have only yielded partial results [62,63,64,65], so this information is not available. Similarly, concerning the YRS and the SRS, there are no official figures for their number either. To overcome this difficulty for the three groups, we followed the procedure of Baruffaldi and Landoni [80], who also had similar difficulties in accessing data from foreign researchers in Italy. The questionnaire was therefore sent to the scientists by the associations and entities supporting the successful pursuit of a research career in Spain. In this way, in the case of the SSA, the following associations and entities have collaborated in the dissemination of the questionnaire: Society of Spanish Researchers in the United Kingdom/Comunidad de Científicos Españoles en el Reino Unido (SRUK/CERU), Científicos Españoles en la República Federal de Alemania (CERFA), Asociación de Científicos Españoles en Japón/Association of Spanish Researchers based in *Japan* (ACE Japón), Españoles Científicos en USA (ECUSA), Asociación de Científicos Españoles en Suecia/Association of Spanish Scientists in Sweden (ACES/FSFS), Spanish Research in Australia-Pacific/Investigadores Españoles en Australia-Pacífico (SRAP/IEAP), Científicos Españoles en Dinamarca/Spanske Forskere i Danmark (CED-SFD), Asociación de Investigadores Españoles en la República Italiana (ASIERI), Red de Científicos Españoles en México (RECEMEX), Asociación de Investigadores Españoles en Irlanda (SRSI), Asociación de Científicos Españoles en Suiza (ACECH),Científicos Españoles en Bélgica/Spanish Scientists in Belgium (CEBE), Sociedad de Investigadores Españoles en Francia/Société de Chercheurs Espagnols en France (SIEF/SCEF), Red de Investigadores China-España/Network of Researchers China-Spain (RICE), Asociación de Investigadores Españoles en Noruega/Spanske Forskere i Norge(IENO/SFNO) and Proyecto Volvemos. The latter Project, together with the following entities and associations, has provided the SRS data: Científicos Retornados a España (CRE), Fundación Universidad-Empresa (FUE), and the Asociación Española para el Avance de la Ciencia (AEAC). Finally, the YRS data have come from the Federación de Jóvenes Investigadores (FJI), Colegio Oficial de Físicos (COFIS), FUE, Federación Española de Biotecnólogos (FEBiotec), ARATECH, Centro de Innovación de la Universidad de Oviedo, and Scientists Dating Forum (Sci-df).

The group of participants compiled with all the data from these associations and entities is made up of 5199 individuals for a sample of 291 subjects finally obtained (YRS: 107; SSA: 124; SRS: 60). In this survey, a maximum sampling error of +5.00% (confidence level: 90.00%) is estimated, which falls within the parameters required for a sample of these characteristics [81]. Furthermore, the percentages of responses obtained are considerably higher (5.60%) than those collected in the GEM Spain Report 2018–2019 [82], from the same period as the survey conducted (0.078%; confidence level: 95.5%; sampling error: +0.64%), in a country like Spain with little activity of scientists in companies and possibly because of this, with a reduced business culture within this group, as will be discussed below.

From 17 December 2018 to 30 April 2019, the associations and entities sent the questionnaire by email to their members. Approximately every two weeks, the survey’s researchers informed the associations and entities of the number of responses obtained so that they could continue to send the questionnaire by email. The last call was sent at the end of April. The survey concluded on 30 April 2019.

### 2.3. Questionnaire: Procedure and Data Analysis

The variables in the questionnaire have been divided into the following blocks:

#### 2.3.1. Block 1. Entrepreneurship Training

As stated above, there is a positive attitude towards entrepreneurship training [23,24,25,26], since it generates positive stimuli and increases the entrepreneurial intention of students [27,28,29]. Therefore, in this questionnaire, the SSA and SRS were asked whether they had received entrepreneurship training in the course of their scientific careers abroad. In the case of the YRS, when pursuing their career in Spain, the question is limited to whether they have received entrepreneurship training in the country. For the remaining variables, the questions are asked in the same way by groups.

#### 2.3.2. Block 2. Acquisition of Entrepreneurial Skills

Earlier, we stressed how important it was for scientists in health areas to acquire entrepreneurial skills to transfer their research results to the market [16]. For this paper, these skills have been adapted from a list (see Table 1) established by Martínez and Carmona [83], due to how they integrate significant aspects of the traditional skill-based approach, linked to the business and professional world, and that of the key skills, the latter more associated with the educational and social field. In this regard, entrepreneurial skills aim to achieve the autonomy of the individual and are geared towards the self-fulfillment of the subject in the pursuit of a life project focused on the production of goods and services that satisfy the needs of the community and that reactivate its economic activity, which is why this approach to skills focuses on such an important aspect of entrepreneurship as the development and consolidation of an entrepreneurial culture.

This approach is in line with the Spanish case, since, according to the latest figures available, among the countries in its closest area, the European Union (EU), Spain is one of the countries with the lowest percentage of researchers in companies [84]. In Spain, over the last decade of available data, this percentage has ranged from 33.70% (2010) to 38.79% (2018), while in the EU it has ranged from 44.69% (2009) to 52.80% (2018), which reveals a significant gap that has increased to 14.00% in recent years. These figures show that the business culture may not be very deep-rooted among Spanish scientists, although these cultural aspects are so decisive in promoting entrepreneurship [85].

A Likert Scale of 1 to 4 points was used to assess these variables, with higher values indicating a higher level of acquisition of skills and lower values at a lower level. As the results indicate, only the percentages in the higher ratings representing the greater acquisition of skills (3) and (4) were taken into account. A Likert scale of 1 to 4 has been chosen to avoid the appearance of central tendency bias. Furthermore, with this type of scale, no adverse effects are observed in the number of alternatives regarding the measurement of statistics such as the mean or variance [86].

#### 2.3.3. Block 3. Entrepreneurial and Intrapreneurial Intentions

The entrepreneurial skills acquired by scientists through international mobility should be reflected in the intention of researchers to set up companies or carry out intrapreneurial projects. In this regard, as stated above, the number of companies created by scientists is low [37,57,58], international mobility being a factor that encourages this entrepreneurship [34,35,36,58] and intrapreneurship [58].

Concerning the questions to be asked in this block, the report issued by the Global Entrepreneurship Monitor (GEM) is of great value because it provides empirical data on entrepreneurship that is comparable both internationally and over time [59], which gives it great credibility, and therefore the definitions set out in its Adult Population Survey have been adapted to ensure the validity of the questionnaire. According to these definitions, in the case of entrepreneurship, researchers were asked whether they intend to start up a new technology-based company in the next 3 years, which is analogous to the GEM concept of potential entrepreneurship [59]. In the case of intrapreneurship, the question asked was whether researchers intended to carry out intrapreneurship in their scientific organization in the next three years. In the particular case of this study, the adapted definition of this concept [59] shows that it is a process carried out by a person who is involved in the leadership and development of an entrepreneurial initiative for the organization in which they work (University, Public Research Organization, company, etc.).

#### 2.3.4. Block 4. Profile of Participants

Similarly, to implement effective training and scientific mobility policies that promote the acquisition and development of entrepreneurial skills, the review of the profile of the researchers who participated in the survey took into account the contribution of Franzoni, Scellato, and Stephan [61], who asked about the possibility of researchers returning in the future with several options: yes, no, it depends on the job opportunities or perhaps part-time or at the end of my career. In our paper, this question is asked identically for SSA. For SRS, the question is whether they intend to return abroad. For YRS, we have tried to find out about the possibility of these researchers going abroad to continue with their research career because this group has not yet moved and the importance of their having a satisfactory research career [87,88]. To complete this profile, we have followed the approach used by Baruffaldi and Landoni [80], for whom the likelihood of researchers returning increases with a more temporary professional situation and with reasons for leaving that are not related to the improvement of job opportunities. According to this survey, other variables also considered were: sex, type of organization, geographical location, and area of knowledge.

To guarantee the quality of the questionnaire, the following steps were taken: (1) selection and definition of the variables, (2) choice of the means of obtaining the researchers’ answers, (3) presentation of the instructions for participants, and (4) conduct of a pilot test with the draft questionnaire.

This pilot test was conducted by a group of ten scientists from different branches of knowledge who were informed of the objectives and variables of the survey. Their answers were necessary to ensure the clarity of the questions and variables and consider the possibility of adding or removing any variable to improve the results. With the information obtained in this pilot test, it was found that no change was needed in the content of the questionnaire.

## 3. Results

### 3.1. Entrepreneurship Training

As shown in Table 2, few YRS in Health Sciences (6 out of 37, 16.22%) have received entrepreneurship training in Spain. This percentage more than doubles (14 out of 37, 37.84%) for YRS in Health Sciences who have received this training abroad. Likewise, for SRS in Health Sciences who have received this training abroad (5 out of 16, 31.25%), the percentage is nearly twice that observed in YRS in Health Sciences. In terms of area of knowledge, these percentages occupy third place for all YRS and the first for the other two groups. About the other areas of knowledge, the high percentage of YRS in Engineering and Architecture that have received training is very striking in this variable.

### 3.2. Acquisition of Entrepreneurial Skills

Given Table 3, the research group in health areas that has occupied first place in the acquisition of entrepreneurial skills most often has been the SRS with 11, followed by the SSA with 4, and finally, the YRS with 2, which shows the importance of scientific mobility for the acquisition of these skills, and this importance will be pursued in the presentation of the results. However, it is also necessary to point out that these results show that in the vast majority of these skills, the acquisition has been high in all groups.

#### 3.2.1. Entrepreneurial Skills Acquired Primarily by SRS in Health Sciences

According to the data in Table 3b, all researchers in this group have acquired the ability to overcome failure (16/16, 100.00%). This skill has also by a high percentage of YRS in Health Sciences: 97.30% (36/37). A high percentage (31/37, 83.78%), although lower than the other groups, of SSA in Health Sciences has also acquired this skill. In terms of position by area of knowledge, these percentages are in the first position for SRS and YRS in Health Sciences and the fifth for SSA in Health Sciences. Concerning the comparison of all areas, high percentages are also shown in all of them, with the following groups standing out for reaching 100.00%: SRS in Health Sciences, Social and Law Sciences and Sciences, SSA in Art and Humanities, and YRS in Social and Law Sciences and Art and Humanities.

With regard to skills based on the observance of a code of ethics, all SRS (16/16, 100.00%) and SSA (37/37, 100.00%) in Health Sciences have acquired this skill (Table 3c). For YRS in this area, this percentage is reduced slightly to 97.29% (36/37). In the percentage comparisons of this skill, it should be noted that high percentages of acquisition of this skill are achieved, with the majority of areas obtaining 100% acquisition of the skill, the SRS and SSA in Health Sciences also achieving this result, as mentioned above.

Likewise, as shown in (Table 3d), a majority of 93.75% (15/16) of SRS in Health Sciences have acquired the skill to conduct meetings. There is a significant reduction in this area in the case of SSA to 75.68% (28/37) and an even greater one in the case of YRS to 64.87% (24/37). In terms of position by area of knowledge, these percentages are in second place for SRS, fourth for YRS, and fifth for SSA. Once again, by area of knowledge and as a whole, there is a majority that reaches high percentages of skill acquisition, with the percentage in SRS in Social and Law Sciences and YRS in Social and Law Sciences and Arts and Humanities standing out in the comparison.

The ability to overcome stress has been widely acquired by the SRS in Health Sciences with a percentage of 93.75% (15/16, See Table 3e). There is also quite a high percentage of over 80.00% for SSA in Health Sciences (30/37) and a somewhat lower one for YRS in this area (28/37, 75.68%). In terms of position by area of knowledge, these percentages are in the first place for SRS, the third for YRS, and the fourth for SSA. As in the above cases, by area of knowledge and as a whole, there is a majority that reaches high percentages of skill acquisition, with the percentage of YRS in Social and Law Sciences and in Arts and Humanities standing out in the comparison.

According to the figures in (Table 3l), almost 94% (15/16) of SRS in Health Sciences have acquired a proactive attitude, which enables them to carry out activities that are essential to this work, such as the introduction of new products, services, or innovative technology. This proportion also reaches a significant figure, of over 80% (30/37), for SSA in Health Sciences. In the last place, although also with a considerable proportion, are YRS in Health Sciences with 78.38% (29/37). In terms of position by area of knowledge, these percentages for the Health Sciences are in fourth place for SRS and fifth for SSA and YRS. In the percentage comparisons by area, high percentages of skill acquisition are again shown, with the following groups standing out: SRS in Social and Law Sciences and in Arts and Humanities and YRS in Social and Law Sciences and Arts and Humanities.

Leadership skills have again been acquired by almost 94.00% (16/17) of SRS in Health Sciences (see Table 3j). This high percentage is reduced to one that is also significant and identical to that of the previous skill (30/37, 81.08%) in the case of SSA in Health Sciences. In YRS in this area, this percentage is further reduced to levels below 60.00% (22/37). The first group occupies second place in terms of the percentage position by area, while SSA are in fourth place and YRS are in fifth place. In the percentage comparison by area, high percentages of skill acquisition are again observed, with the percentage of skill acquisition for all scientific groups in Social and Law Sciences and YRS in Arts and Humanities standing out.

Almost 70% (11/16) of the SRS in Health Sciences have acquired negotiating skills (see Table 3l). This percentage drops sharply to 51.36% (19/37) in the case of YRS in Health Sciences, although the drop is even greater for SSA in this area, falling to 37.84% (14/37). About position by area of knowledge, these percentages are in the first position for SRS in Health Sciences, and the fifth for SSA, and the fourth for YRS in this area. In terms of the percentage comparison by area, lower percentages were observed than in the previous cases, with YRS in Art and Humanities standing out as the highest at 77.78%.

As shown in (Table 3ll), 87.50% (14/16) of SRS in Health Sciences have acquired organization and delegation skills. This percentage is reduced to a lower, but still significant, rate of 78.37% (29/37) for YRS in the same area. The rate for SSA in Health Sciences is also significant, but lower than for the two previous groups (28/37, 75.67%). In terms of position by area of knowledge for these groups in Health Sciences, SRS are in second place, SSA in fourth, and YRS in third. The percentage comparison by area once again shows high percentages of skill acquisition in most groups and areas, with the 100.00% obtained by SRS in Social and Law Sciences standing out.

A high percentage of 81.25% (13/16) of SRS in Health Sciences has acquired the skill of selecting personnel (see Table 3n). This percentage is reduced to 75.68% (28/37) in the case of SSA in Health Sciences. This reduction is higher in YRS in Health Sciences, falling to 57.03% (21/37). In terms of area of knowledge, these percentages for the Health Sciences are in second place for SRS, third for SSA, and fifth for YRS. In the percentage comparisons by area, somewhat lower percentages of skill acquisition are shown than in most of the previous cases, with some, such as SRS in Engineering and Architecture, being well below 50.00% (16.67%, to be precise), although others, such as SRS in Social and Law Sciences, reach the highest level of 100.00%.

In terms of perseverance, all SRS in Health Sciences have acquired this skill (16/16, 100.00%) (see Table 3o). This level is also almost achieved by SSA in the same area (36/37, 97.30%). This percentage decreases slightly for YRS in Health Sciences to 91.90% (34/37). This last percentage occupies third place in the classification by areas for this skill, while those for SRS and SSA reach higher positions: first and second, respectively. With regard to the percentage comparison by area of knowledge, it should also be pointed out that the percentages are very high in general, with the following groups standing out for obtaining a rate of 100.00%: SRS in Health Sciences and in Social and Law Sciences, and SSA and YRS in Art and Humanities.

Finally, the skills acquired to a greater extent by the group of SRS in Health Sciences (see Table 3p) include the ability to have the foresight and a project for the future, in which this group leads the rest of the groups, with 81.25% (13/16). YRS in Health Sciences follow the foregoing group at 72.98% (27/37). SSA in Health Sciences closes this classification of the area with a slight reduction in comparison with the previous group (26/37, 70.27%). With regard to the position by area of knowledge, these percentages for Health Sciences occupy the second place for SRS and fourth place for both SSA and YRS. In terms of the percentage comparison by areas, high percentages of skill acquisition are shown, with the following groups standing out for reaching the figure of 100.00%: SRS in Social and Law Sciences, and SSA in the same area and in Art and Humanities.

#### 3.2.2. Entrepreneurial Skills Acquired to a Greater Extent by SSA in Health Sciences

In addition to the skill of following a code of ethics described above, in which SSA in Health Sciences achieve a position of shared leadership with SRS in this area, other skills in which they achieve this position are those described below, starting with that related to a positive mental attitude. A high rate of 86.49% (32/37) of this group has acquired this skill, which is higher than the 81.25% (13/16) of SRS in Health Sciences that have also acquired this skill, and the 75.68% (28/37) of YRS in Health Sciences with the same level of skill acquisition. In terms of position by area of knowledge, these percentages are in second place for SSA in Health Sciences, and fourth for the other two groups in the same area. The percentage comparison by areas once again shows high percentages of skill acquisition, with those obtained by SRS and YRS in Social and Law Sciences standing out at 100.00%.

The skill of ease of social relations has been acquired by almost 90.00% (33/37) of SSA in Health Sciences (see Table 3f). This high percentage is reduced slightly or both SRS in Health Sciences (13/16, 87.50%) and YRS in the same area (28/37, 86.49%). The first group and the third group occupy third place in terms of position by area of knowledge, while the second group is in second place. In the percentage comparison by areas, high percentages of skill acquisition are obtained, with YRS in Social and Law Sciences and in Art and Humanities standing out at 100.00%.

According to (Table 3h), a high rate of over 91.00% (34/37) of SSA in Health Sciences has acquired conversation skills. This percentage falls, although not excessively, to 87.50% (14/16) in the case of SRS in Health Sciences. For YRS in this area, the percentage falls even further to 81.08% (30/37). In relation to positions by area of knowledge, these percentages are in third place for the three groups in Health Sciences. In terms of the percentage comparison by areas, as in most of the previous skills, high percentages are observed, with the following groups standing out at 100.00%: SRS in Social and Law Sciences, SSA in Art and Humanities, and YRS in both Social and Law Sciences and Art and Humanities.

The latter group also reached a leading position in the skill of motivating employees with a percentage of 94.59% (35/37). This high percentage is reduced to some extent in the case of SRS in the same area (14/16, 87.50%). This reduction is greater for YRS in Health Sciences, who achieve a rate of 70.27% (26/37) for this skill. The first two percentages are in second place in the classification by area for this skill, while the percentage for YRS is in fourth place. With regard to the percentage comparison by area of knowledge, it should also be pointed out that the percentages are quite high in general, with the following groups standing out with a rate of 100.00%: SRS and SSA in Social and Law Sciences and YRS in Art and Humanities.

#### 3.2.3. Entrepreneurial Skills Acquired to a Greater Extent by YRS in Health Sciences

In this group, leadership positions are only achieved in two skills: time management and planning. With regard to the former (see Table 3g), the skill has been acquired by a large majority of the group’s members (35/37, 94.60%). This very high percentage is significantly reduced in the next group, that of the SRS in Health Sciences (12/16, 75.00%), and even more so in SSA in Health Sciences (26/37, 70.27%). With regard to the position by area of knowledge, the first percentage, that of YRS, is in second place, being in first place for SRS in Health Sciences and in second place for SSA in the same area. The percentage comparison by area shows lower percentages of skill acquisition than in many of the previous cases, with the very low rate of 16.67% obtained by SRS in Engineering and Architecture standing out. In this regard, the top rate of 100% obtained by YRS in Arts and Humanities is also noteworthy.

With regard to planning, as can be seen in (Table 3m), this skill has been acquired by all YRS in Health Sciences (37/37, 100.00%). This rate is reduced to a still significant percentage of 93.75% (15/16) in the case of SRS in Health Sciences and a considerably high rate of 91.90% (34/37) for SSA in the same area. By area of knowledge, these percentages are in first, second, and third place, respectively. In terms of the percentage comparison by area, very high percentages of skill acquisition are achieved for all of them, with rates of 100.00% being obtained in three areas by the YRS group (Health, Social and Law Sciences, and Art and Humanities) and in Social and Law Sciences by SRS.

### 3.3. Entrepreneurial and Intrapreneurial Intentions

Concerning the area of Health Sciences (see Table 4), the group with the most intentions in this respect are SRS (entrepreneurial intentions: 2/16, 12.50%; intrapreneurial intentions: 8/16, 50.00%), followed by SSA (entrepreneurial intentions: 2/37, 5.41%; intrapreneurial intentions: 12/37, 32.43%), and finally, YRS (entrepreneurial intentions: 0/37, 0.00%; intrapreneurial intentions: 10/37, 27.03%), it being noteworthy that there is no researcher from this group with entrepreneurial intentions.

With regard to the position by area of knowledge, in the case of entrepreneurial intentions, these percentages put SRS in Health Sciences in second place and the other two groups in the same area in fourth and fifth place. For intrapreneurial intentions in this area of Health Sciences, this classification puts the SRS in the first place, the YRS in second, and the SSA in third.

In the percentage comparison by area, the data provided by the GEM for the same period (2018–2019) will be taken into account [81]. The potential entrepreneurship figure for Spain is 6.8%, occupying the last position among 31 high-income countries. The average rate for these countries is 20.4%, for EU28 countries it is 14.1%, and the country with the highest rate of potential entrepreneurship among these high-income countries is Chile with 50.2%. According to these figures, among the Health Sciences groups, only SRS exceed the Spanish average and comes close to the average of the EU28 countries. It is also worth noting that, in general, the mobile SRS (11.67%) and SSA (8.87%) groups do exceed the Spanish potential entrepreneurship rates. Other groups and areas which stand out in this respect are as follows: SRS in Engineering and Architecture (33.33%), SSA in Social and Law Sciences (28.57%), and YRS in Engineering and Architecture (25.00%).

In the case of intrapreneurship, although the figures are not comparable, as the GEM 2018–2019 [81] shows the actual figures of this rate, while this paper provides figures on intentions, they can serve as a reference for decision-making by the health authorities. Among high-income countries, data from this GEM 2018–2019 [81] show an intrapreneurship rate for Spain of 1.7% and an average rate for the EU28 countries of 5.2%, while the countries with the highest intrapreneurship rate are Canada and Ireland with 8.6%. All these data are amply exceeded by those of the groups and areas considered, with the exception of the cases of YRS and SRS in Social and Law Sciences, and the high rates shown in SRS in Health Sciences (50.00%) and SSA in Arts and Humanities (42.86%).

### 3.4. Profile of Participants

**Figure 1:**
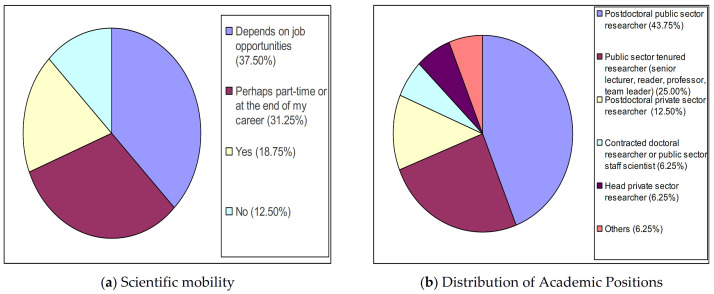
Profile of health sciences scientists returned to Spain. (**a**) Scientific mobility; Health Sciences will not go abroad again to carry out scientific work. (**b**) Distribution of Academic Positions.

Figure 1a shows that 12.50% (2/16) of SRS in Health Sciences will not go abroad again to carry out scientific work. On the other hand, the percentages of scientists from this group who are sure they will go abroad again (18.75%, 3/16) or who will leave the country depending on job opportunities (37.50%, 6/16) are much higher. According to Figure 1b, the percentage of SRS in Health Sciences who work in permanent jobs in the scientific career is lower than that of those who work in temporary jobs. As shown in Figure 1b, the total figure for permanent jobs reaches a percentage of 37.50% (6/16), once the figures for contracted doctoral researchers or public sector staff scientists (6.25%, 1/16), tenured researchers (25.00%, 4/16) and head private-sector researchers (6.25%, 1/16) are added.

**Figure 2:**
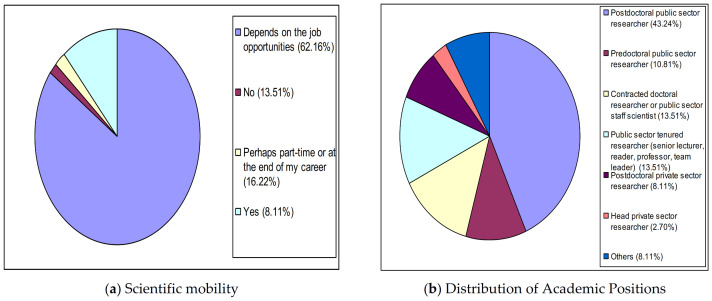
Profile of health sciences spanish scientists abroad. (**a**) Scientific mobility. (**b**) Distribution of Academic Positions.

According to Figure 2a, just 13.51% (5/37) of the Health Sciences SSA group is sure that they will not return to Spain to carry out scientific work, while the percentage of those who might return to Spain depending on job opportunities is very high (62.16%, 23/37), to which can be added the percentage of those who are sure they will return (8.11%, 3/37). As with the aforementioned group (see Figure 2b), although with a greater gap, the total figure for temporary jobs (70.27%, 79/147) also significantly exceeds that of permanent jobs (29.73%, 11/37) consisting of contracted doctoral researchers or public sector staff scientists (13.51%, 5/37), tenured researchers (13.51%, 5/37) and head private-sector researchers (2.70%, 1/37).

**Figure 3:**
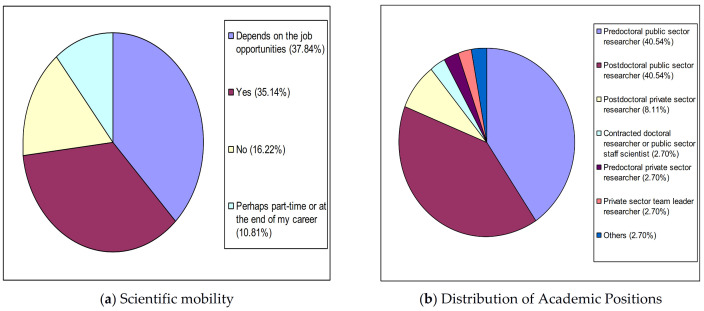
Profile of health sciences young researchers in Spain. (**a**) Scientific mobility. (**b**) Distribution of Academic Positions.

With regard to the profile of YRS in Health Sciences, as shown in Figure 3a, 16.22% (6/37) are sure that they will not go abroad to continue carrying out scientific work. This percentage is lower than both the 35.14% (13/37) of those who intend to go abroad and the 37.84% (14/37) of those who may leave the country depending on job opportunities. Also, in Figure 3b, it can be seen to an even greater extent than that shown in the previous cases, that the total figure for temporary jobs (94.60%, 35/37) is much higher than that of permanent jobs consisting of contracted doctoral researchers or public sector staff scientists (2.70%, 1/37) and head private-sector researchers (2.70%, 1/37), which is only 5.40% (2/37).

As can be seen in the figures, these data are replicated for the overall sample. In these figures, data are also provided for all the knowledge areas and the total sample of the other variables considered in the profile of the participants in this survey: gender, type of organization, geographical location, and area of knowledge.

## 4. Discussion

### 4.1. Discussion of the Research Results

As shown in the results, few YRS in Health Sciences have received training in entrepreneurship in Spain. On the other hand, both YRS in Health Sciences and SRS in the same area show better figures for training in entrepreneurship abroad. However, these results are also rather poor and do not conform to the positive view of entrepreneurship training described above [23,24,25,26]. Nevertheless, this positive view *is* greater abroad, so to grow in this area, it is necessary for the health authorities to promote scientific mobility, in addition to trying to increase entrepreneurship training in Spain.

With regard to the acquisition of entrepreneurial skills, SRS in Health Sciences have been the most outstanding research group and have positioned themselves in first place in the greatest number of these entrepreneurial skills, followed by SSA in Health Sciences, and finally, YRS in the same area, which shows the importance of scientific mobility for the acquisition of these skills.

It should also be pointed out that the acquisition of the large majority of these skills has been high in all groups. Only the group of YRS in Health Sciences show low figures, but even these are greater than 50% for the skills of negotiation, recruitment, and leadership. For SSA in this area, such figures are only recorded for the skill of negotiation, which drops as low as less than 50%. This is also the case with SRS in Health Sciences, in which group the negotiating skill is the least widely acquired, although with rates of much more than 50%. Therefore, it is desirable to focus much of the training on the negotiation issue, which affects all three groups, although it would also be advisable to concentrate some training on recruitment and leadership, which primarily affect YRS in Health Sciences.

However, this high level of skill acquisition is not reflected in a high degree of entrepreneurial intentions on the part of the Health Science groups involved in scientific mobility, something which is consistent with the small number of companies that such scientists create [37,57,58]. However, these intentions are greater in the groups that have carried out this mobility, such as SRS and SSA, especially the former and in the case of intrapreneurship, which is also in line with what has been explained above regarding these issues [34,35,36,58]. These results show that the lack of training in entrepreneurship shown above is reflected in a reduced intention to create companies as the positive stimuli referred to by the authors cited above are not created [27,28,29].

It is quite possible that the high acquisition of skills will be focused on intrapreneurship, given the much higher intention rates shown by these scientific groups in this respect, which is very important to help a country like Spain climb positions in global intrapreneurship within high-income economies [82], and which may also allow these researchers to transfer their results to the market [16], especially taking into account the results of the aforementioned survey carried out by the World Economic Forum (WEF) in collaboration with the Global Entrepreneurship Monitor (GEM) [60], which showed that countries with more intrapreneurs create more employment and competitiveness than those with more entrepreneurs. However, it is not desirable for the rates of entrepreneurial intention to be low or non-existent, as is the case of YRS in Health Sciences considered in this survey, so given that this group has received the least training in this respect, it is quite likely that training in these subjects could be of great value in promoting this entrepreneurial intention and in the creation of companies in general.

In terms of comparison with other areas of knowledge, the results have been quite similar to those obtained for the Health Sciences. In this regard, the groups in this health area achieved good positions, with the SRS standing out, with two important leaderships: training in entrepreneurship among returned scientists, and intrapreneurial intentions for all the groups in the sample. With regard to data from other areas, the high percentage of YRS in Engineering and Architecture who have received training in entrepreneurship is particularly noteworthy. As for the acquisition of entrepreneurial skills, the groups of CRE and YRS in Social and Law Sciences, and YRS in Arts and Humanities also obtained ratings of 100% in many of them. Similarly, in terms of entrepreneurial intentions, groups such as SRS in Engineering and Architecture, SSA in Social and Law Sciences, and YRS in Engineering and Architecture stand out. Finally, in the case of intrapreneurial intentions, the high percentages of SSA in Arts and Humanities stand out but are nonetheless exceeded by SRS in Health Sciences, as mentioned above. Given these results, it might be useful to study the measures carried out in these outstanding groups and areas for these variables to see if they could give rise to any good practices applicable to the Health Sciences.

With regard to SRS in Health Sciences, the percentage of those with a permanent job is low, while in terms of scientific mobility, the percentage of those who will go abroad again depending on job opportunities is the highest. Similarly, the percentage of SSA in Health Sciences who have a permanent job is quite low and the percentage of scientists from this group who might return to Spain depending on job opportunities is the highest. The percentage of YRS in Health Sciences with a permanent job is also very low, the highest being those who would leave Spain to continue carrying out scientific work depending on job opportunities.

In accordance with this profile for these three groups, and what has been explained above [61,80], the creation of job opportunities becomes a key part of scientific mobility, as has been highlighted in various surveys carried out for the health area [74,75], so the health authorities could achieve adequate scientific mobility if they provided the conditions for such job opportunities to be created or created the job opportunities directly.

According to the data obtained on entrepreneurial intentions, the creation of companies is hardly considered a job opportunity by the Spanish Health Science scientific groups involved in scientific mobility, especially YRS. To increase these intentions and the creation of companies, policies must be established to activate training in entrepreneurial subjects or to promote scientific mobility itself, which, as we have shown in the paper, provides greater training experience in entrepreneurship.

With regard to intrapreneurial intentions, this activity can be considered as a job opportunity by these scientific groups, as it is clearly related to the idea of the creation of entrepreneurial hospitals, as explained above [5,9,10,11], in addition to the opportunities of this nature that may arise in other scientific organizations such as Universities or Public Research Bodies and companies in the health sector. As shown in the results, the rates achieved are considerable, especially in the case of the mobile groups of SRS and SSA, as was the case for entrepreneurial intentions, so policies for collaboration with these groups must be intensified through the associations and entities that represent them. Through this collaboration, which, as mentioned above, is not excessive [76], Spanish healthcare organizations and companies related to the sector can access innovative ideas that lead to lines of business and transfer to the market and society, obtain new funding channels, and enter new markets due to the knowledge provided by scientific groups abroad regarding the mindset of these countries. These groups can even help YRS to carry out their mobility abroad so that, to the benefit of all, they can open up new possibilities for international collaboration with advanced countries, which have a greater capacity to mobilize economic and human resources and attract new partners [89].

As these collaborations progress, these scientific organizations and companies must offer health science researchers stable job opportunities to encourage them to return or stay on once they have returned. In this regard, some papers [74] highlight a series of key measures that could encourage return, such as stable funding, salary, recognition of scientific careers, and the implementation of Social Responsibility in the organization they work for. Suitable working conditions and personal benefits could also be established to encourage this return, as shown in other papers [75]. A key organization for the application of these measures of a social nature would be the universities through their Social Responsibility for their capacity to produce a positive impact on society through higher education, research, knowledge transfer, and sustainability [90,91]. Within these measures of Social Responsibility of Universities, it would be convenient to include the collaboration and monitoring of their former scientists who work abroad, as some cases show how all the scientists who returned had maintained ties with their organization of origin [91].

As has been observed, aspects such as the scant training in entrepreneurship provided to scientists, or the reduced entrepreneurial intentions on their part, especially for the group of scientists who have not carried out mobility abroad, have been ratified in this work, but a novel contribution has also been made concerning the important generation of intrapreneurial intentions, especially in the group of scientists with mobility abroad, which together with the other novel contribution about the high acquisition of entrepreneurial skills, provides relevant data to the health authorities so that within their policies to attract scientific knowledge, they try to identify these intrapreneurial researchers with mobility abroad through the associations of scientists abroad and returnees exposed in this work, in such a way that these scientists Spaniards can lead knowledge transfer projects in the health area that make the NHS sustainable. For all this, the information contained in this paper has implications for the improvement of national public health services, as appropriate training and scientific mobility policies that encourage entrepreneurship and intrapreneurship in these services can boost innovation and transfer so that the results obtained can be transferred and contribute to economic and social development.

### 4.2. Limitations and Future Lines of Research

The data provided to support the conclusions of this work are reliable and consistent with other research but show the limitation of having been obtained from a small number of Spanish scientists in the Health Sciences. To ratify the validity of these conclusions in future papers, an attempt will be made to increase this figure.

Similarly, the lack of knowledge about the total population of the three groups of researchers studied has made it necessary to use data from different associations and institutions, so the results obtained cannot be extrapolated to all the scientists in these groups. Furthermore, this paper has assessed entrepreneurship training, the acquisition of entrepreneurial skills, and the intentions of researchers involved in scientific mobility in Health Sciences, but not their progress, so in future papers, the timeframe will be extended with a longitudinal design. Likewise, the results of this research are an invitation to continue exploring the relationship between training, scientific mobility, and entrepreneurship, especially on the qualitative plane, to promote research into achieving greater knowledge transfer in the Health Sciences.

## 5. Conclusions

For all the above reasons, this paper has provided data that shows that mobile scientists in the Health Sciences are more entrepreneurial and intrapreneurial, have acquired more entrepreneurial skills, and have received more education in these subjects. Therefore, both training and scientific mobility encourage entrepreneurship and, in particular, intrapreneurship, and are measures that the health authorities can intensify to promote innovation and knowledge transfer for the benefit of greater economic and social development.

## Figures and Tables

**Table 1:** Types of entrepreneurial skills to be acquired and their description.

Entrepreneurial Skills	Description
Positive mental attitude	Building subjects’ confidence in their own abilities and skills so that they can successfully develop their own business/enterprise.
Ability to overcome failure	Stressing the difficulty of carrying out a business project, motivating and training employees so that they do not drop out of the project as soon as the first difficulties or adversities arise but continue trying.
Code of ethics	Entrepreneurial skills must be based on ethics, so entrepreneurs must understand that all their actions and behavior will be governed by moral rules based on respect for other people and nature.
Meeting management	Developing the necessary knowledge and skills to smoothly run meetings with future employees, and other commercial, political, economic, or social agents.
Stress management	Training subjects to control themselves in stressful situations.
Social relations	Interacting easily and appropriately with other people in different situations and contexts
Time management	Organizing time properly in the activities carried out daily.
Conversation skills	Ability to communicate fluently and speak appropriately with other people in different situations or contexts.
Proactive attitude	Doing things for yourself, promoting initiatives, introducing new products, services, or innovative technology.
Leadership	Promoting the ability to direct and guide, with respect for others, the future employees of your business or company.
Employee motivation	Ability to encourage future employees to perform their work with interest.
Negotiation	Ability to trade (e.g., buy and sell or exchange goods or merchandise to increase the profit of the company).
Organization and delegation	Being competent in coordinating all your employees and dividing up the different tasks and functions of your business.
Planning	Organizing your company/business/intrapreneurship plan in an orderly, coherent, and pragmatic way.
Personnel selection	Ability to choose the workers who will be part of your company appropriately.
Perseverance	Learning to be constant with a task or action and not to give up before finishing it.
Foresight and project for the future	Generating ideas and identifying opportunities that have not been seen by other employers.

**Table 2:** Entrepreneurship training.

Area of Knowledge	Researcher Groups (%) ^a^		
	YRS ^b^ Entrepreneurship Training in Spain	SSA ^c^ Entrepreneurship Training Abroad	SRS ^d^ Entrepreneurship Training Abroad
Health Sciences	16.22%	37.84%	31.25%
Social and Law Sciences	20.00%	28.57%	0.00%
Arts and Humanities	11.11%	28.57%	25.00%
Sciences	12.50%	35.09%	17.86%
Engineering and Architecture	43.75%	37.50%	16.67%
Total	18.69%	35.48%	21.67%

^a^ Percentage of researchers who have received entrepreneurship training. ^b^ Young researchers in Spain. ^c^ Spanish scientists abroad. ^d^ Scientists returned to Spain.

**Table 3:** The magnitude of the acquisition of entrepreneurial skills by area of knowledge and research group.

	**(a) Positive Mental Attitude**			**(b) Ability to Overcome Failure**		
**Area of Knowledge**	**Researcher Groups (%) ^a^**			**Researcher Groups (%) ^a^**		
	**YRS ^b^**	**SSA ^c^**	**SRS ^d^**	**YRS ^b^**	**SSA ^c^**	**SRS ^d^**
Health Sciences	75.68%	86.49%	81.25%	97.30%	83.78%	100.00%
Social and Law Sciences	100.00%	85.71%	100.00%	100,00%	85.71%	100,00%
Arts and Humanities	88.89%	85.71%	87.50%	100,00%	100,00%	75,00%
Sciences	80.00%	82.46%	89.28%	87.50%	85.97%	100.00%
Engineering and Architecture	62.50%	93.75%	66.67%	75.00%	87.50%	83.33%
Total	77.57%	85.49%	85.00%	90.66%	86.29%	95.00%
	**(c) Code of Ethics**			**(d) Meeting Management**		
**Area of knowledge**	**Researcher Groups (%) ^a^**			**Researcher Groups (%) ^a^**		
	**YRS ^b^**	**SSA ^c^**	**SRS ^d^**	**YRS ^b^**	**SSA ^c^**	**SRS ^d^**
Health Sciences	97.29%	100.00%	100.00%	64.87%	75.68%	93.75%
Social and Law Sciences	100.00%	85.71%	100.00%	100.00%	85.71%	100.00%
Arts and Humanities	100.00%	100.00%	87.50%	100.00%	85.71%	87.50%
Sciences	100.00%	96.50%	100.00%	60.00%	82.46%	89.28%
Engineering and Architecture	93.75%	100.00%	100.00%	75.00%	93.75%	66.67%
Total	98.13%	97.58%	98.33%	69.16%	82.25%	83.33%
	**(e) Stress Management**			**(f) Social Relations**		
**Area of knowledge**	**Researcher Groups (%) ^a^**			**Researcher Groups (%) ^a^**		
	**YRS ^b^**	**SSA ^c^**	**SRS ^d^**	**YRS ^b^**	**SSA ^c^**	**SRS ^d^**
Health Sciences	75.68%	81.08%	93.75%	86.49%	89.99%	87.50%
Social and Law Sciences	100.00%	85.71%	50.00%	100.00%	85.71%	50.00%
Arts and Humanities	100.00%	57.14%	62.50%	100.00%	100.00%	87.50%
Sciences	72.50%	61.41%	85.71%	82.50%	77.19%	96.43%
Engineering and Architecture	75.00%	93.75%	50.00%	81.25%	93.75%	66.67%
Total	77.57%	72.90%	80.00%	85.98%	84.67%	88.33%
	**(g) Time Management**			**(h) Conversation Skills**		
**Area of knowledge**	**Researcher Groups (%) ^a^**			**Researcher Groups (%) ^a^**		
	**YRS ^b^**	**SSA ^c^**	**SRS ^d^**	**YRS ^b^**	**SSA ^c^**	**SRS ^d^**
Health Sciences	94.60%	70.27%	75.00%	81.08%	91.89%	87.50%
Social and Law Sciences	80.00%	85.71%	50.00%	100.00%	85.71%	100.00%
Arts and Humanities	100.00%	71.43%	50.00%	100.00%	100.00%	87.50%
Sciences	77.50%	64.91%	60.72%	72.50%	82.46%	100.00%
Engineering and Architecture	75.00%	87.50%	16.67%	75.00%	93.75%	66.67%
Total	85.04%	70.97%	58.33%	79.44%	87.90%	91.67%
	**(i) Proactive Attitude**			**(j) Leadership**		
**Area of knowledge**	**Researcher Groups (%) ^a^**			**Researcher Groups (%) ^a^**		
	**YRS ^b^**	**SSA ^c^**	**SRS ^d^**	**YRS ^b^**	**SSA ^c^**	**SRS ^d^**
Health Sciences	78.38%	81.08%	93.75%	59.46%	81.08%	93.75%
Social and Law Sciences	100.00%	85.71%	100.00%	100.00%	100.00%	100.00%
Arts and Humanities	100.00%	85.71%	100.00%	100.00%	71.43%	87.50%
Sciences	80.00%	87.72%	100.00%	70.00%	84.21%	85.71%
Engineering and Architecture	81.25%	93.75%	83.33%	81.25%	87.50%	83.33%
Total	82.24%	86.29%	93.33%	71.97%	83.87%	88.33%
	**(k) Employee Motivation**			**(l) Negotiation**		
**Area of knowledge**	**Researcher Groups (%) ^a^**			**Researcher Groups (%) ^a^**		
	**YRS ^b^**	**SSA ^c^**	**SRS ^d^**	**YRS ^b^**	**SSA ^c^**	**SRS ^d^**
Health Sciences	70.27%	94.59%	87.50%	51.36%	37.84%	68.75%
Social and Law Sciences	80.00%	100.00%	100.00%	60.00%	71.43%	50.00%
Arts and Humanities	100.00%	85.71%	87.50%	77.78%	71.43%	50.00%
Sciences	72.50%	82.46%	82.14%	62.50%	59.65%	57.15%
Engineering and Architecture	56.25%	93.75%	83.33%	43.75%	68.75%	66.67%
Total	71.96%	88.71%	85.00%	53.27%	55.65%	60.00%
	**(ll) Organization and Delegation**			**(m) Planning**		
**Area of knowledge**	**Researcher Groups (%) ^a^**			**Researcher Groups (%) ^a^**		
	**YRS ^b^**	**SSA ^c^**	**SRS ^d^**	**YRS ^b^**	**SSA ^c^**	**SRS ^d^**
Health Sciences	78.37%	75.67%	87.50%	100.00%	91.90%	93.75%
Social and Law Sciences	60.00%	71.43%	100.00%	100.00%	85.71%	100.00%
Arts and Humanities	77.78%	85.71%	75.00%	100.00%	100.00%	75.00%
Sciences	85.00%	80.70%	71.43%	85.00%	85.97%	75.00%
Engineering and Architecture	93.75%	100.00%	50.00%	93.75%	93.75%	83.33%
Total	82.24%	81.45%	85.00%	93.46%	89.52%	81.67%
	**(n) Personnel Selection**			**(o) Perseverance**		
**Area of knowledge**	**Researcher Groups (%) ^a^**			**Researcher Groups (%) ^a^**		
	**YRS ^b^**	**SSA ^c^**	**SRS ^d^**	**YRS ^b^**	**SSA ^c^**	**SRS ^d^**
Health Sciences	57.03%	75.68%	81.25%	91.90%	97.30%	100.00%
Social and Law Sciences	80.00%	85.71%	100.00%	80.00%	85.71%	100.00%
Arts and Humanities	77.78%	71.43%	50.00%	100.00%	100.00%	87.50%
Sciences	65.00%	57.89%	71.43%	95.00%	85.96%	100.00%
Engineering and Architecture	68.75%	87.50%	16.67%	87.50%	100.00%	83.33%
Total	64.49%	69.35%	66.67%	92.52%	91.93%	96.66%
	**(p) Foresight and Project for the Future**					
**Area of knowledge**	**Researcher Groups (%) ^a^**					
	**YRS ^b^**	**SSA ^c^**	**SRS ^d^**			
Health Sciences	72.98%	70.27%	81.25%			
Social and Law Sciences	80.00%	100.00%	100.00%			
Arts and Humanities	88.89%	100.00%	62.50%			
Sciences	72.50%	68.42%	78.58%			
Engineering and Architecture	75.00%	93.75%	50.00%			
Total	74.77%	75.81%	75.00%			

^a^ Percentage of researchers who have indicated a “significant” or “very significant” acquisition of entrepreneurial skills. ^b^ Young researchers in Spain. ^c^ Spanish scientists abroad. ^d^ Scientists returned to Spain.

**Table 4:** Entrepreneurial and intrapreneurial intentions by area of knowledge and research group.

**Area of Knowledge**	**Research Groups with Entrepreneurial Intentions (%) ^a^**		
	**YRS ^b^**	**SSA ^c^**	**SRS ^d^**
Health Sciences	0.00%	5.41%	12.50%
Social and Law Sciences	0.00%	28.57%	0.00%
Arts and Humanities	0.00%	0.00%	12.50%
Sciences	2.50%	10.53%	7.14%
Engineering and Architecture	25.00%	6.25%	33.33%
Total	4.67%	8.87%	11.67%
**Area of Knowledge**	**Research Groups with Intrapreneurial Intentions (%) ^e^**		
	**YRS ^b^**	**SSA ^c^**	**SRS ^d^**
Health Sciences	27.03%	32.43%	50.00%
Social and Law Sciences	0.00%	28.57%	0.00%
Arts and Humanities	33.33%	42.86%	37.50%
Sciences	12.50%	22.81%	32.14%
Engineering and Architecture	18.75%	37.50%	33.33%
Total	19.63%	29.03%	36.67%

^a^ Percentage of researchers with entrepreneurial intentions. ^b^ Young researchers in Spain. ^c^ Spanish scientists abroad. ^d^ Scientists returned to Spain. ^e^ Percentage of researchers with intrapreneurial intentions.

## Data Availability

The data from this study are shown in the tables and in the Supplementary Materials.
